# Effect of Vaccination on Platelet Mitochondrial Bioenergy Function of Patients with Post-Acute COVID-19

**DOI:** 10.3390/v15051085

**Published:** 2023-04-28

**Authors:** Anna Gvozdjáková, Jarmila Kucharská, Zuzana Rausová, Guillermo Lopéz-Lluch, Plácido Navas, Patrik Palacka, Barbora Bartolčičová, Zuzana Sumbalová

**Affiliations:** 1 Pharmacobiochemical Laboratory of 3rd Medical Department, Faculty of Medicine, Comenius University in Bratislava, Sasinkova 4, 811 08 Bratislava, Slovakia; 2 Centro Andaluz de Biologia del Desarrollo, Instituto de Salud Carlos III, Universidad Pablo de Olavide—CSIC-3A and CIBERER, 41013 Seville, Spain; 3 2nd Department of Oncology, Faculty of Medicine, Comenius University in Bratislava, 811 08 Bratislava, Slovakia; 4 Faculty of Civil Engineering, Slovak Technical University, 811 07 Bratislava, Slovakia

**Keywords:** SARS-CoV-2 virus, post-acute COVID-19, vaccination, platelets, mitochondrial oxidative phosphorylation, coenzyme Q10, antioxidants, lipid peroxidation

## Abstract

Background: Mitochondrial dysfunction and redox cellular imbalance indicate crucial function in COVID-19 pathogenesis. Since 11 March 2020, a global pandemic, health crisis and economic disruption has been caused by SARS-CoV-2 virus. Vaccination is considered one of the most effective strategies for preventing viral infection. We tested the hypothesis that preventive vaccination affects the reduced bioenergetics of platelet mitochondria and the biosynthesis of endogenous coenzyme Q_10_ (CoQ_10_) in patients with post-acute COVID-19. Material and Methods: 10 vaccinated patients with post-acute COVID-19 (V + PAC19) and 10 unvaccinated patients with post-acute COVID-19 (PAC19) were included in the study. The control group (C) consisted of 16 healthy volunteers. Platelet mitochondrial bioenergy function was determined with HRR method. CoQ_10_, γ-tocopherol, α-tocopherol and β-carotene were determined by HPLC, TBARS (thiobarbituric acid reactive substances) were determined spectrophotometrically. Results: Vaccination protected platelet mitochondrial bioenergy function but not endogenous CoQ_10_ levels, in patients with post-acute COVID-19. Conclusions: Vaccination against SARS-CoV-2 virus infection prevented the reduction of platelet mitochondrial respiration and energy production. The mechanism of suppression of CoQ_10_ levels by SARS-CoV-2 virus is not fully known. Methods for the determination of CoQ_10_ and HRR can be used for monitoring of mitochondrial bioenergetics and targeted therapy of patients with post-acute COVID-19.

## 1. Introduction

On 11 March 2020, the World Health Organization (WHO) declared a global pandemic, health crisis, and economic disruption caused by SARS-CoV-2 virus, responsible for a new type of acute respiratory infection with atypical pneumonia. The disease was named COVID-19 (Corona Virus Disease 2019) [1]. In December 2022, there were approximately 641 million cases of COVID-19 and 6 million deaths due to COVID-19 worldwide [2].

In many patients, symptoms of COVID-19 persist for several weeks to months. Signs of COVID-19 can be divided into two groups: as subacute COVID-19 (post-acute COVID-19), including signs present for 4–12 weeks after acute COVID-19 and chronic (post-COVID-19 syndrome) including symptoms over 12 weeks after the SARS-CoV-2 infection [3].

The main clinical symptoms of COVID-19 include an elevated temperature above 37 °C, or a high temperature of 38–42 °C; chills; dry cough; shortness of breath; difficulty breathing at rest or during exercise; headache; muscle and joint pain; general weakness; hair loss; loss of taste, smell, and hearing; sleep and memory disturbances; depression; and impairment of quality of life [4]. Frequent complications of COVID-19 include cardiomyopathy, arrhythmias, thrombosis, pulmonary embolism, and multiorgan failure [5].

Several mechanisms participate in SARS-CoV-2 infection. Dysfunction of the mitochondrial and immune system are key factors in COVID-19 in aging [6,7,8,9]. The virus may modulate antiviral immunity signaling, alter the intracellular distribution of mitochondria, induce platelet dysfunction and aggregation, increase oxidative stress, and reduce antioxidant protection [10]. In 2020 we hypothesized that mitochondria and endogenous coenzyme Q_10_ (CoQ_10_) biosynthesis may be the targets of the SARS-CoV-2 virus [11], and two years later we documented platelet mitochondrial bioenergy dysfunction, reduced endogenous CoQ_10_ level and oxidative stress in patients with post-COVID-19 syndrome [12,13].

The ACE2 receptor has a dual role, it mediates the interaction between host cells and SARS-CoV-2 spike protein and regulates the renin-angiotensin system for the cardiovascular and immune systems [14]. Other mechanisms of action of the SARS-CoV-2 virus independent of the ACE2 receptor are assumed. It is proposed that the SARS-CoV-2 virus interacts with platelets and megakaryocytes [15] and that the SARS-CoV-2 spike protein binds to platelets through CD42b receptor. This link explains the hypercoagulation, the triggering of monocyte activation, and cytokine storm in severe disease of COVID-19 [16].

The SARS-CoV-2 virus also manipulates mitochondrial energy production directly by localizing the viral open reading frame 9b (ORF-9b) protein to the outer mitochondrial membrane of the host. The result of this interaction is the disruption of mitochondrial antiviral signaling and suppression of innate immunity [17]. ORF9b protein interacts with mitochondrial import receptor subunit TOM70 by suppressing interferon expression. A recent study has shown that SARS-CoV-2 encodes nine accessory proteins that may also contribute to immune system evasion: ORF3a, ORF3b, ORF6, ORF7a, ORF7b, ORF8, ORF9b, and two proteins ORF9c, ORF10 [18]. An in vitro study has shown a novel mechanism by which SARS-CoV-2 inhibits the innate immune response through ORF10 that induces mitophagy-mediated degradation of mitochondrial antiviral signaling protein (MAVS) [19]. Other studies have demonstrated that the SARS-CoV-2 virus hijacks the host mitochondria of immune cells in COVID-19 [20]. Mitochondrial hijacking by the SARS-CoV-2 virus could be a key factor in the pathogenesis of this virus and the COVID-19 induction [21]. SARS-CoV-2 reprograms mitochondrial metabolism in peripheral blood mononuclear cells and in monocytes of patients with COVID-19 towards energy production by glycolysis instead of mitochondrial oxidative phosphorylation [22,23]. SARS-CoV-2 virus can also target intracellular and extracellular mitochondria [20], which play a central role in the primary host defense mechanisms against viral infections. Platelet aggregation is one of the important characteristics of COVID-19, with an increased risk for thrombosis development. In the host cell, viruses regulate Ca^2+^ homeostasis, block mitochondrial metabolic pathways such as the β-oxidation of fatty acids and the Krebs cycle, and generate oxidative stress, which causes mitochondrial redistribution [24].

The importance of endogenous CoQ_10_ biosynthesis for immune response and reducing the severity of SARS-CoV-2 virus infection has been published [12,25]. CoQ_10_ is an integral part of the mitochondrial respiratory chain in the inner membrane and a key substance for ATP production. CoQ_10_ exists in mitochondrial Q-CYCLE (fully oxidized—ubiquinone), fully reduced (ubiquinol), and semiquinone radical (ubisemiquinone). Ubiquinol is considered one of the strong endogenous antioxidants, which prevents or reduces lipid peroxidation [26].

CoQ_10_ as a mobile electron carrier accepts electrons from NADH through Complex I (NADH ubiquinone oxidoreductase) and/or from FADH_2_ through Complex II (succinate dehydrogenase) to cytochrome c through CoQH_2_-cytochrome c reductase (Complex III). Cytochrome c transfers the electrons to oxygen through Complex IV (cytochrome c oxidase). Protons are pumped to the mitochondrial intermembrane space and proton motive force is produced, which is used for ATP synthesis at Complex V (by ATP synthase) via oxidative phosphorylation [12,27].

It is believed that the most effective strategy to prevent COVID-19 infection is vaccination [28]. The first of the vaccines preventing COVID-19 for administration to humans was produced by Moderna Inc. on 16 March 2020 [29]. BionTech/Pfizer and Moderna vaccines contain genetic information messenger RNA (mRNA) for the formation of the spike protein, with which SARS-CoV-2 enters the cells of the body. During vaccination against the SARS-CoV-2 virus, the mRNA-lipid particles enter muscle cells. The immune system recognizes the spike protein as foreign, initiates a defensive response and protective antibodies begin to form. Astra Zeneca vaccine belongs to the group of vector vaccines. The basis of vector vaccines is a non-pathogenic virus, a chimpanzee adenovirus having the function of a carrier of genetic information. After the vaccine application, the gene of the SARS-CoV-2 virus enters the cells of the human body, and spike protein is produced. The immune system identifies this protein as foreign and begins to create antibodies [29,30].

Repeated doses of vaccines (boosters) are applied to improve prevention and immunity against COVID-19. Despite this, many people become infected with the SARS-CoV-2 virus with a milder course of the disease. SARS-CoV-2 virus affects older as well as younger age groups, and patients with comorbidities such as cardiovascular and nephrology disease, hypertension, obesity, diabetes mellitus, chronic respiratory system disease, and oncological disease [31,32,33]. Based on available literature data, the effects of preventive vaccination against the SARS-CoV-2 virus on platelet mitochondrial bioenergetics and endogenous coenzyme Q_10_ biosynthesis have not been published. We tested the hypothesis that preventive vaccination affects the bioenergetics of platelet mitochondria and endogenous coenzyme Q_10_ biosynthesis in patients with post-acute COVID-19.

## 2. Materials and Methods

### 2.1. Study Design and Patients

Two groups of patients with post-acute COVID-19 were included in this study: Vaccinated patients with post-acute COVID-19 (V + PAC19) 2 weeks after infection and unvaccinated patients with post-acute COVID-19 (PAC19) 4–7 weeks after infection.

#### 2.1.1. Control Group (C)

The control group consisted of 16 healthy volunteers (7 men and 9 women), aged 38–67 years, mean age of 43.0 ± 3.1 years. Inclusion criteria: no history of COVID-19, absence of chronic medication, without coenzyme Q_10,_ and without vitamin E supplementation. Exclusion criteria: diseases, obesity, smoking, and regular alcohol consumption.

#### 2.1.2. Vaccinated Patients with Post-Acute COVID-19 (V + PAC19) 2 Weeks after Infection

In October 2021 in Bratislava, Slovakia, 10 vaccinated people (5 men and 5 women), aged 18–55 years with a mean of age 41.5 ± 3.8 years were together at a party and all became infected with the SARS-CoV-2 virus. Two weeks after overcoming acute COVID-19 without hospitalization, blood samples of these non-infectious patients were collected for examination.

Baseline characteristics of patients: Five patients vaccinated twice with the Astra Zeneca vaccine were infected with the SARS-CoV-2 virus two months after vaccination. Four patients vaccinated with the BionTech/Pfizer vaccine were infected with the SARS-CoV-2 virus 2–3 months after the second vaccination. One patient vaccinated with the Moderna vaccine was infected with the SARS-CoV-2 virus one month after vaccination.

Clinical symptoms were recorded by a (patient) questionnaire before their inclusion in the study. The main clinical symptoms during the acute COVID-19 included general fatigue (9/10), muscle pain (5/10), joint pain (6/10), headache (6/10), shortness of breath (1/10), pressure on the chest (3/10), a burning sensation in the lungs (2/10), pain between shoulder blades (1/10), cough (5/10), increased temperature 37–37.8 °C (4/10), fever above 38 °C (3/10), chills (3/10), sneezing (2/10), runny nose (2/10), expectoration of mucus (2/10), weight loss (1/10), loss of appetite, loss of smell (3/10), nausea (2/10), dizziness (1/10), sleep disorders (2/10), eye pain (1/10).

Treatment of patients after vaccination as recommended by doctors: Agen (1/10), Duloxetine (1/10), Aerius (1/10), Cosentyx (1/10), Nolpaza (1/10), Isoprinosine (3/10), Sumamed (1/10), Lyndinette (1/10). The daily doses of vitamins were the following: 500–1000 mg of vitamin C, vitamin D (1000–2000 IU), Zn, Se (1/10); B12, Zn + vitamin C, vitamin D3 + K2 (1/10); lactobacillus (1/10). Patients had a mild course of the acute illness of COVID-19 and at the time of blood sampling, all were working with minimal persistent symptoms.

#### 2.1.3. Unvaccinated Patients with Post-Acute COVID-19 (PAC19) 4–7 Weeks after Infection

In January–February 2021 in Bratislava, Slovakia, we examined 10 patients in the PAC19 group (3 men and 7 women), aged 41–81 years with an average age of 59.1 ± 4.2 years. Patients were cured at home without hospitalization, they had a mild to moderate course of COVID-19. Blood sampling was performed 4.7 ± 0.4 weeks after acute COVID-19.

Clinical symptoms were recorded by a (patient) questionnaire before their inclusion in the study. The main persistent clinical symptoms included general fatigue (7/10), weakness, dry cough (1/10), impaired breathing during exercise (2/10), and loss of appetite accompanied with significant weight loss (2/10, 7, and 10 kg) [34].

Treatment of patients as recommended by doctors: Daily dose 1000–2000 mg of vitamin C, 2000–4000 IU of vitamin D3, 50–100 mg Zn. Two patients also used vitamin K2, two patients were treated with antibiotics (Sumamed), and 1 patient with Isoprinosine. During the laboratory examination, the patients were non-infectious [34].

### 2.2. Methods

#### 2.2.1. Platelet Preparation

Platelets were isolated from whole blood [35,36] as described previously [37]. The platelet suspension was counted on the hematological analyzer Mindray BC-6200 (Mindray, Shenzhen, China).

#### 2.2.2. High-Resolution Respirometry Method

Mitochondrial bioenergetics in platelets was evaluated by the high-resolution respirometry (HRR) method with the use of an O2k-Respirometer (Oroboros Instruments, Innsbruck, Austria) [38,39]. For the evaluation of platelet mitochondrial bioenergetics, substrate-uncoupler-inhibitor (SUIT) protocol 1 was applied [40] as described in detail previously [12].

#### 2.2.3. High-Performance Liquid Chromatography (HPLC)—Determination of CoQ_10-TOTAL_, α-tocopherol, γ-tocopherol, β-carotene

Total coenzyme Q_10_ concentration (ubiquinol + ubiquinone) in whole blood, plasma, and isolated platelets was determined by HPLC [41,42]. For the oxidation of ubiquinol to ubiquinone, 1,4-benzoquinone was added to the sample. Concentrations of analyzed substances were calculated in µmol/L and pmol/10^9^ PLT [43,44].

#### 2.2.4. TBARS

Thiobarbituric acid reactive substances (TBARS) as a parameter of oxidative stress—an indicator of lipid peroxidation, were determined in plasma by spectrophotometric method [45].

#### 2.2.5. Data Analysis

The results in graphs and tables are expressed as mean ± standard error of the mean (sem). Unpaired Student’s *t*-test was applied to evaluate the difference between the parameters of the control group and patient groups with post-acute COVID-19. *p* values < 0.05 were considered statistically significant. Differences in platelet mitochondrial bioenergetics, coenzyme Q_10_, and γ- and α-tocopherol in vaccinated and unvaccinated patients with post-acute COVID-19 are expressed in percentages compared to the control values taken as 100%.

The study was carried out according to the principles expressed in the Declaration of Helsinki, and the study protocol was approved by the Ethics Committee of Academic Derer’s Hospital in Bratislava, Slovakia, No. EK/012/2021/UNB. This study is registered by ClinicalTrials.gov ID: NCT05178225. Written informed consent form was obtained from each subject before the start of the study.

## 3. Results

### 3.1. The Effect of Vaccination on the Platelet Mitochondrial Bioenergy Function in Patients with Post-Acute COVID-19 (V + PAC19)

**Figure 1:**
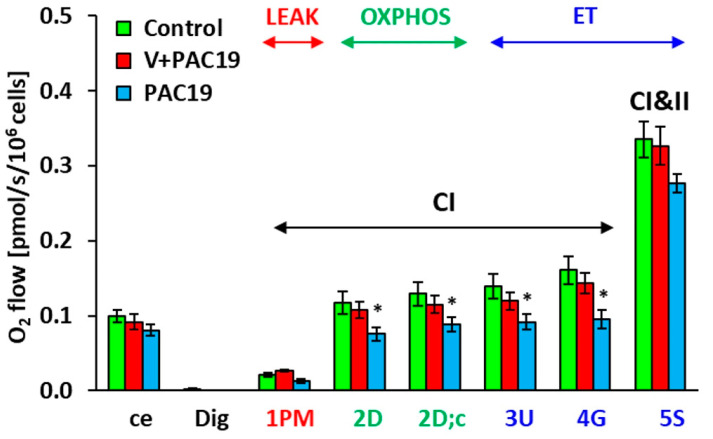
Platelet mitochondrial respiration in vaccinated and unvaccinated patients with post-acute COVID-19 (V + PAC19, PAC19) was evaluated as O_2_ flow [pmol/s/10^6^ cells]. Legends: The parameters are named according to the titration steps in the SUIT protocol and represent respiration after the corresponding titration step: ce—intact cells (platelets); Dig—digitonin; 1 PM—pyruvate plus malate; 2D—adenosine diphosphate (ADP); 2D; c—cytochrome c; 3U—uncoupler FCCP; 4G—glutamate; 5S—succinate. The bars show mean ± sem. LEAK—non-phosphorylating resting state of respiration; OXPHOS—the phosphorylating state of respiration associated with ATP production; ET—noncoupled state of respiration at an optimum concentration of uncoupler. CI—complex I pathway; CI and II—complex I and complex II pathway. Control—control group (n = 16); V + PAC19—vaccinated patients with post-acute COVID-19 (n = 10); PAC19—unvaccinated patients with post-acute COVID-19 (n = 10); * *p* < 0.05—statistical significance vs. control group.

All measured parameters of platelet mitochondrial function are evaluated as O_2_ flow [pmol/s/10^6^ cells]. Parameters of platelet mitochondrial function of vaccinated patients with post-acute COVID-19 (V + PAC19) were not significantly changed in comparison with control data. Control values are taken as 100%. Routine respiration of intact platelets (ce) reached 92.7% ± 10.8% vs. control values. In permeabilized platelets, mitochondrial oxygen consumption (State 4) with Complex I-linked substrates (1 PM) was slightly not significantly stimulated to 123.0% ± 7.3% in comparison with control data. Complex I-linked OXPHOS respiration coupled with ATP production (2D) slightly decreased to 92.1% ± 9.3%, respiration after cytochrome c addition (2D;c) slightly decreased to 88.8% ± 8.9%, maximal mitochondrial oxidative capacity (the electron transfer capacity, ET) after uncoupler titration (3U) reached 85.5% ± 8.1% of control group value, the respiration after addition of CI-linked substrate glutamate (4G) reached 89.2% ± 8.4% of control data, the ET capacity with CI + CII-linked substrates (5S) reached 97.3% ± 7.5% of control group values (Figure 1, Table 1).

### 3.2. The Effect of SARS-CoV-2 Virus on the Platelet Mitochondrial Bioenergy Function in Unvaccinated Patients with Post-Acute COVID-19 (PAC19)

**Figure 2:**
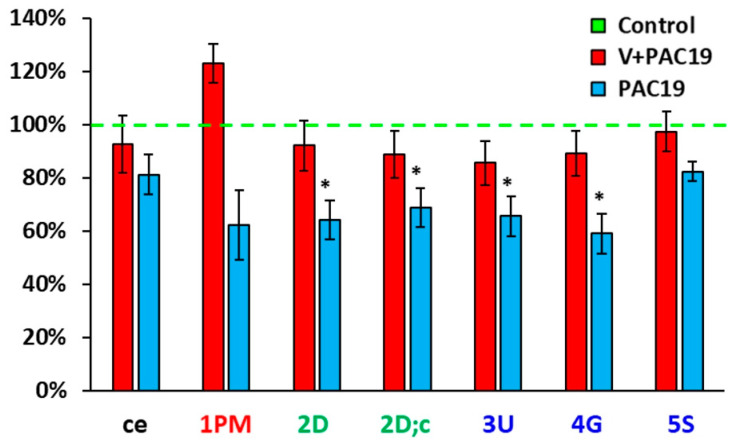
Differences of platelet mitochondrial bioenergetics in % between control group, vaccinated (V + PAC19) and unvaccinated patients (PAC19) with post-acute COVID-19. Legends: see Figure 1, * *p* < 0.05—statistical significance vs. control group.

The effect of the SARS-CoV-2 virus on the platelet mitochondrial bioenergy function in unvaccinated patients 4.7 ± 0.4 weeks after acute COVID-19 (PAC19) is shown in Figure 1, Table 1. Routine respiration (ce) of intact platelets of patients with PAC19 reached 81.2% ± 7.5% of control data values. In platelets permeabilized with digitonin, mitochondrial oxygen consumption (State 4) with Complex I-linked substrates (1 PM) reached 62.4% ± 13.0% of control values. CI-linked respiration coupled with ATP production (2D)—(CI-linked OXPHOS) significantly (*p* = 0.034) decreased to 64.1% ± 7.4%, the respiration after cytochrome c addition (2D;c) decreased (*p* = 0.026) to 68.7% ± 7.4%, maximal mitochondrial oxidative capacity (ET capacity) evaluated after uncoupler titration (3U) significantly decreased (*p* = 0.039) to 65.6% ± 7.4% of control data. The respiration after the addition of CI-linked substrate glutamate (4G) significantly decreased (*p* = 0.020) to 59.1% ± 7.4% of control data. The ET capacity with CI + CII-linked substrates (5S) reached 82.4% ± 3.7% of control values (Figure 1 and Figure 2, Table 1) [35]. SARS-CoV-2 virus significantly reduced the platelet mitochondrial Complex I-linked bioenergy function coupled with ATP production in patients 4–7 weeks with post-acute COVID-19 (PAC19), (Figure 1).

Figure 2 shows differences in platelet mitochondrial bioenergetics between vaccinated and unvaccinated patients with post-acute COVID-19. Parameters are evaluated in % vs. control group, which is taken as 100%. While in unvaccinated patients of the PAC19 group (Figure 1 and Figure 2, Table 1) Complex I-linked respiration and ATP production was significantly decreased, in the vaccinated group (V + PAC19) these parameters are preserved. Therefore, we suppose that vaccination may protect platelet mitochondrial Complex I-linked respiration and ATP production from SARS-CoV-2 virus damage (Figure 1 and Figure 2, Table 1).

### 3.3. The Effect of SARS-CoV-2 Virus on the Concentration of CoQ_10-TOTAL_ in Platelets, Blood, Plasma and TBARS in Vaccinated and Unvaccinated Patients with Post-Acute COVID-19 (V + PAC19, PAC19)

**Figure 3:**
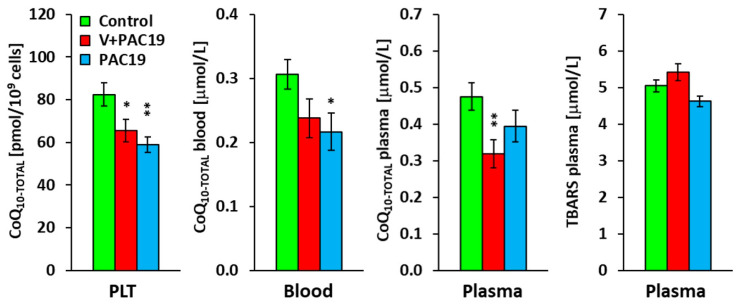
The concentration of CoQ_10-TOTAL_ in platelets, blood, plasma, and TBARS in vaccinated and unvaccinated patients with post-acute COVID-19 (V + PAC19, PAC19). Legends: Control—control group; V + PAC19—vaccinated patients with post-acute COVID-19; PAC19—unvaccinated patients with post-acute COVID-19; CoQ_10-TOTAL_ (ubiquinol + ubiquinone); PLT—platelets; TBARS—thiobarbituric acid reactive substances; The bars show mean ± sem. * *p* < 0.05, ** *p* < 0.01—statistical significance vs. control group.

Vaccination did not prevent the deficit of endogenous CoQ_10_ in platelets of patients with post-acute COVID-19. CoQ_10-TOTAL_ concentration was significantly lower in platelets (*p* = 0.046), and reached 80 ± 6% compared to control data; in whole blood, the concentration reached 78 ± 10% (*p* = 0.085), and in plasma, the concentration was significantly lower (*p* = 0.010), reaching 67 ± 8% of control values. TBARS was slightly, not significantly, increased in the V + PAC19 group of patients to 107 ± 5% vs. the control group (Figure 3, Table 2).

A deficit of CoQ_10_ was found in unvaccinated patients with post-acute COVID-19. CoQ_10-TOTAL_ concentration in platelets reached 71 ± 5% of the control group value in the PAC19 group (*p* = 0.004). CoQ_10-TOTAL_ in PAC19 group blood reached 71 ± 10% (*p* = 0.031), and CoQ_10-TOTAL_ in plasma reached 83 ± 10% of the control group. We did not find statistically significant differences between CoQ_10-TOTAL_ concentrations of vaccinated and unvaccinated patients. In the PAC19 group, TBARS was slightly decreased to 92 ± 5% compared to control data (Figure 3, Table 2).

### 3.4. The Effect of the SARS-CoV-2 Virus on the Antioxidants in Platelets, Blood, and Plasma in Vaccinated and Unvaccinated Patients with Post-Acute COVID-19 (V + PAC19, PAC19)

Vaccination also affected the levels of other antioxidants in patients in the V + PAC19 group. In platelets the concentration of γ-tocopherol was significantly decreased (*p* = 0.051), reaching 51 ± 10% of control data; whereas in unvaccinated patients (PAC19) platelet levels of γ-tocopherol did not suffer any decrease reaching a 110 ± 19% vs. controls (*p* = 0.9). The concentration of α-tocopherol was slightly increased to 124 ± 17% of the control group in vaccinated patients in comparison with a clear decrease found in unvaccinated patients reaching 37 ± 5% (*p* = 0.0001) of control levels.

In whole blood of vaccinated patients, concentrations of γ-tocopherol were 80 ± 18%, α-tocopherol 90 ± 9%, and β-carotene 81 ± 11% of control concentrations. On the contrary, unvaccinated patients suffered a higher decrease in all the levels: γ-tTocopherol (68 ± 10%), α-tocopherol (80 ± 9%), and β-carotene (64 ± 12%) of control data.

**Figure 4:**
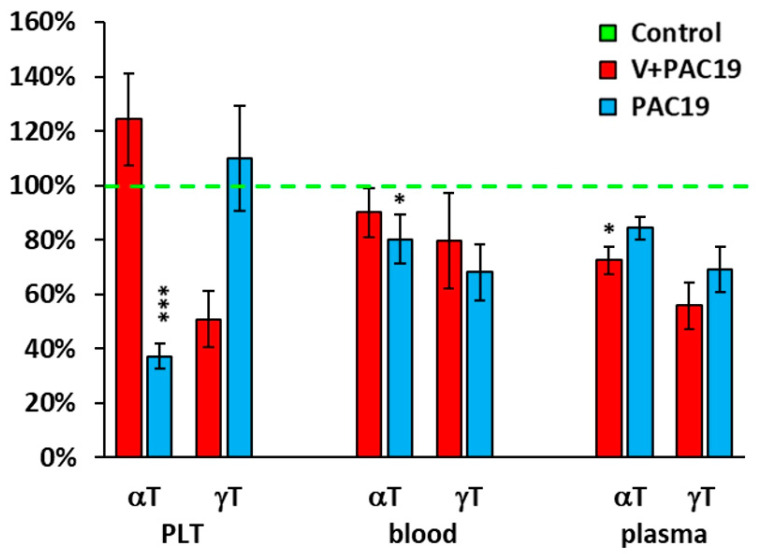
Endogenous α- and γ-tocopherols levels in platelets, whole blood, and plasma in vaccinated and unvaccinated patients with post-acute COVID-19 as % of control group values. Legends: Control—control group; V + PAC19—vaccinated patients with post-acute COVID-19; PAC19—unvaccinated patients with post-acute COVID-19; PLT—platelets, αT—α-tocopherol, γT—γ-tocopherol. Results are expressed in percentages, and control group values are taken as 100%. * *p* < 0.05, *** *p* < 0.001—statistical significance vs. control group.

In plasma of vaccinated patients, the concentration of γ-tocopherol reached 56 ± 9% (*p* = 0.067), α-tocopherol reached 72 ± 5% (*p* = 0.021), and β-carotene increased to 111 ± 17% of controls levels (Figure 4, Table 2). In this case, unvaccinated patients did not show significant differences; γ-tocopherol reached 69 ± 8%, α-tocopherol reached 84 ± 4% and β-carotene increased to 126 ± 25% of control levels (Figure 4, Table 2).

## 4. Discussion

The SARS-CoV-2 virus infected hundreds of million people in the world and caused about 18.2 million deaths until the end of 2021 [46]. New strategies for COVID-19 prevention and therapy were used, including regular exercise in rehabilitation, physical activity, and pharmacologic and antioxidant therapies [47,48,49]. Based on many reviewed trials, the use of lipophilic vitamins A, D, and E in the prevention and treatment of patients suffering from SARS-CoV-2 acute infection seems reasonable [50]. Micronutrient supplementation is recommended in patients with post-COVID-19 syndrome [51]. Vitamin E and its components α- and γ-tocopherol have reactive oxygen species (ROS) scavenging potential. They protect cells from oxidative damage, increase the integrity of cell membranes, and improve the adaptive response of the immune system to viral infections [52]. Mountain spa rehabilitation and the simultaneous effect of mountain spa rehabilitation together with ubiquinol supplementation improved platelet mitochondrial Complex I-linked oxidative phosphorylation and pulmonary function of patients with post-COVID-19 syndrome [12,13].

In this study, we compared the effect of SARS-CoV-2 virus infection on platelet mitochondrial bioenergy function, coenzyme Q_10_, antioxidants and oxidative stress on vaccinated and unvaccinated patients with post-acute COVID-19.

The SARS-CoV-2 virus is a single-stranded positive-sense RNA β-coronavirus, which mutates with a high speed. Known mutations are Alpha, Beta, Gamma, Delta, and Omicron [53]. The SARS-CoV-2 genome encodes four structural proteins including the spike (S), membrane (M), envelope (E), and nucleocapsid (N) proteins. The non-structural proteins NSP1-NSP15 and accessory proteins are included in the SARS-CoV-2 virus function [54,55]. SARS-CoV-2 spike proteins are responsible for the initial host receptor recognition, their attachment to the angiotensin-converting enzyme 2 (ACE2) present in nose and mouth cells, and pass through the mucous membranes of the nose, larynx, and airways to the lungs [56]. The SARS-CoV-2 virus may suppress innate immunity, modulate mitochondrial bioenergetic function, produce a “cytokine storm”, cause mitochondrial dysfunction, a deficit of ATP production, and a reduction of cell protection by antioxidants [17]. In 2022 we confirmed that after SARS-CoV-2 virus infection, Complex I-linked platelet mitochondrial respiratory chain function and endogenous CoQ_10_ level were reduced in patients with post-COVID-19 syndrome [12].

Preventive and therapeutic strategies for the infection of COVID-19 were developed. Vaccination is the most effective preventive strategy against SARS-CoV-2 virus infection. In 2021, patients in our study (V + PAC19 group) were vaccinated with BionTech/Pfizer (Comirnaty), Astra-Zeneca (Vaxzervia), and Moderna (Spikevax) vaccines, but were nevertheless infected with the SARS-CoV-2 virus. These vaccines prevented declines in platelet mitochondrial respiration and energy production as shown in patients 2 weeks after acute infection with the SARS-CoV-2 virus (Figure 2, Table 1). Measured parameters of platelet mitochondrial function were slightly, but not significantly decreased in comparison with the control group. Complex I-linked respiration (1 PM), Complex I-linked OXPHOS capacity (2D, 2D;c), and ET-capacity with CI + CII-linked substrates (5S) did not significantly differ from control data. We suppose that vaccination may protect platelet mitochondrial bioenergetics by several mechanisms: by reduction of inflammatory signaling in megakaryocytes [19,57] and by blocking the entry of the SARS-CoV-2 virus into the blood and into cells [57]. An alternative mechanism of infection by the SARS-CoV-2 virus, independent of the ACE2 receptor, is the binding of the spike protein of SARS-CoV-2 to platelets via the CD42b receptor [16]. During vaccination against the SARS-CoV-2 virus, the immune system recognizes the entry of a foreign spike protein into the body, and antibodies against the spike protein begin to form. Vaccination prevents entry of SARS-CoV-2 into platelets because the antibodies bind to the spike protein and thus the virus cannot bind to ACE2 or CD42b receptors. Therefore, mitochondrial function is protected [16]. We have shown that platelet mitochondrial bioenergetics of previously vaccinated patients examined 2 weeks after acute COVID-19 were preserved (Figure 2, Table 1), whereas in unvaccinated patients, the CI-linked OXPHOS was severely impaired.

Complexes of the mitochondrial respiratory system are organized into supercomplexes called respirasomes, which offer a shorter distance for electron transfer between complexes. It has been recently demonstrated that CoQ molecules can be used in two pathways in supercomplexes. For the CI + CIII supercomplex, CoQ is exclusively used for the oxidation of NADH (CoQNADH pool) and for Complex II, CoQ is used as a cofactor (CoQFADH pool) [27,58]. SARS-CoV-2 virus’s effect on mitochondrial function can induce reverse electron transfer (RET) from CoQ to NAD^+^, and cellular energy metabolism can be reprogrammed towards increased glycolysis. Complex I could be oxidatively damaged by the accumulation of superoxide anion, and CoQNADH and Complex III are released from supercomplex CI + CIII. This partial release of the CoQNADH pool is then used for the supply of the CoQFADH pool by reprogramming cellular metabolism [58,59]. Mitochondrial dysfunction and energy deficit in PBMC of patients with COVID-19 were compensated by increased glycolysis and RET in the mitochondrial respiratory system [23]. This metabolic manipulation by SARS-CoV-2 triggers an enhanced inflammatory response that contributes to the severity of symptoms in patients with COVID-19. We suppose that vaccination prevents the reprogramming of cellular metabolism by preserving OXPHOS function in vaccinated patients with post-acute COVID-19 (V + PAC19).

In this study, unvaccinated patients (PAC19) were not protected against SARS-CoV-2 virus effects. SARS-CoV-2 virus reduced platelet mitochondrial Complex I-linked respiration to 61.9% (*p* = 0.073) in comparison with the control group. Complex I-linked ADP-stimulated mitochondrial respiration associated with ATP production was reduced in platelets of post-COVID-19 patients to 63.6%, and significantly (*p* = 0.034), mitochondrial respiration after cytochrome c addition was significantly decreased to 69.0% (*p* = 0.026) in comparison with the control group. The deficit in CI-linked OXPHOS in unvaccinated patients with PAC19 can induce reverse electron transfer from CoQ_10_ to Complex I [60,61].

A deficit of endogenous CoQ_10_ biosynthesis is one of the main causes of muscle weakness and fatigue in patients with post-COVID-19 syndrome, and reduced mitochondrial function can contribute to COVID-19 progression [12,16]. In unvaccinated patients 4–7 weeks after PAC19 and in patients 3–6 months after post-COVID-19 syndrome, reduced endogenous CoQ_10_ levels in whole blood, plasma, and platelets were found [13,61]. SARS-CoV-2 virus reduced endogenous CoQ_10_ antioxidant levels in vaccinated and unvaccinated patients with post-acute COVID-19 in platelets, blood, and plasma (Figure 3). The viral protein PDB 6Y84 protease of SARS-CoV-2 seems to be an excellent target receptor for CoQ_10_ [62]. By this interaction, CoQ_10_ may form a direct bond with the main protease of the SARS-CoV-2 virus inhibiting viral replication. The endogenous levels of CoQ_10_ in patients with post-acute COVID-19 were significantly reduced. The exact mechanisms for the depletion of CoQ_10_ during SARS-CoV-2 infection remain to be determined [62]. The decline in endogenous CoQ_10_ levels and in the mitochondrial bioenergetics in platelets were found also in our previous study in patients with post-COVID-19 syndrome. Mitochondrial bioenergetics of platelets recovered after 30 days of supplementation with high doses of ubiquinol combined with mountain spa rehabilitation [12,13]. Viral infections may modulate antioxidant systems and induce ROS production. SARS-CoV-2 virus spike protein enhances platelet ROS levels and aggregation [63]. There are indications that oxidative stress and the defense against ROS are crucial in COVID-19 pathogenesis [64].

In addition to the reduced endogenous CoQ_10_ biosynthesis, decreased antioxidant capacity in patients with COVID-19, as alterations in the activity of glutathione peroxidase, total antioxidant capacity was observed [65,66]. In another study, the opposite results were found. Antioxidant capacity was similar in hospitalized patients with severe and moderate COVID-19 [67]. Levels of antioxidant capacity may depend on the course of the disease. The differences between reported antioxidant capacity in COVID-19 patients could be caused by a smaller sample size which can lead to statistically non-significant differences between analyzed groups [68].

Other antioxidants were also modulated by the SARS-CoV-2 virus of vaccinated or unvaccinated patients with post-acute COVID-19 (Figure 4). We found decreased concentrations of α-tocopherol in platelets in unvaccinated patients but comparable (slightly higher) with the controls in vaccinated patients. On the contrary, concentrations of γ-tocopherol were lower in vaccinated patients, and concentrations in unvaccinated patients were similar to the controls. In plasma, we found a significantly lower concentration of α-tocopherol in vaccinated patients which may be related to its incorporation into platelets, to protect them against oxidative damage. The concentration of γ-tocopherol in plasma was slightly lower in vaccinated patients when compared to healthy controls. In unvaccinated patients, there were no significant differences in plasma α- and γ-tocopherol compared to controls (Figure 4).

Among tocopherols, γ-tocopherol has unique biological properties that α-tocopherol does not have. It is able to trap deleterious nitrogen radicals, inhibit cyclooxygenase-2 (COX-2) activity, and has anti-inflammatory properties. γ-Tocopherol is metabolized by a cytochrome P450-dependent process in the liver, whose activity is inhibited by interleukin and other proinflammatory cytokines. Thus, the metabolism of γ-tocopherol may be altered under oxidative stress [49]. Other authors found that circulatory γ-tocopherol concentrations are directly associated with systemic oxidative stress and inflammation [69]. This may be in agreement with our results when we assume that vaccinated patients had a lower incidence of post-COVID-19 symptoms, and probably lower inflammation, which could result in a reduction of γ-tocopherol concentration. We found no differences in tocopherol concentrations in whole blood between groups. Beta-carotene (β-Car), provitamin A is the most important carotenoid required for transformation to vitamin A in the human body. Vitamin A plays a vital role in regulating immune response and reducing susceptibility to infections, its supplementation prior to infection and during recovery may be beneficial [68]. In our study, we did not find any significant differences in concentrations of β-Car in plasma between vaccinated and unvaccinated patients although they were slightly higher than in controls. Similarly, there were no differences in concentrations of β-Car in whole blood between groups.

The roles of vaccination and supplementation with micronutrients, vitamins A, D, and Zn in post-COVID symptoms were studied [51]. Full vaccination against COVID-19 prevents the disease and the development of residual symptoms in SARS-CoV-2 infection. Vitamins have significant roles in immunity and their supplementation is recommended as part of the therapy. However, we did not find any study focused on concentrations of the lipophilic vitamin E forms (α- and γ-tocopherol) in patients with post-COVID-19 syndrome or on the effect of vaccination on the status of these vitamins.

Oxidative stress markers were found in patients with COVID-19. Increased lipid peroxidation was observed in COVID-19 patients compared to controls [70,71]. Lage et al. 2022 [72] found increased lipid peroxidation and higher mitochondrial superoxide levels in circulating monocytes in patients with mild, moderate, and severe COVID-19 in comparison with the control group. In hospitalized patients, TBARS had similar levels, independent of disease severity [73]. In our study groups, we did not find statistically different values of TBARS concentration in comparison with the control group. In vaccinated patients with post-acute COVID-19, TBARS in plasma was slightly higher, and in unvaccinated patients with post-acute COVID-19, slightly lower in comparison with control data (Figure 4). An imbalance between the free oxygen radical production and antioxidant defense systems plays an important role in the pathogenesis of COVID-19.

## 5. Conclusions

Vaccination against SARS-CoV-2 virus infection prevented the decline of platelet mitochondrial respiration and ATP production via the OXPHOS pathway, associated with complex I of the respiratory system of patients with post-acute COVID-19 2 weeks after infection. In unvaccinated patients with post-acute COVID-19 4–7 weeks after infection, platelet mitochondrial bioenergetics was significantly reduced. Vaccination may protect mitochondrial bioenergetics through several mechanisms, independent of the ACE2 receptor, such as reduction of inflammatory signaling in megakaryocytes [18], or binding of the SARS-CoV-2 spike protein to platelets via the CD42b receptor [19]. In vaccinated and unvaccinated patients with post-acute COVID-19, a reduction of endogenous level/biosynthesis of CoQ_10_ by the SARS-CoV-2 virus was found. The mechanism of suppression of CoQ_10_ level by the SARS-CoV-2 virus is not fully known. Our results contribute to elucidating the mechanism of the positive effect of vaccination on mitochondrial energy production in patients with post-acute COVID-19. Methods for the determination of CoQ_10_ and high-resolution respirometry can be used for monitoring mitochondrial bioenergetics and for targeted therapy of patients with post-COVID-19.

Limitations of the study: The low number of patients in both groups after COVID-19 is the main limitation of our study. Both groups of patients were formed from our circle of acquaintances. Unfortunately, we did not have access to other patients after acute COVID-19. However, in our previous study with a larger number of patients with post-COVID-19 syndrome (n = 14) and (n = 22) [12,13], we found impaired mitochondrial bioenergetics in platelets and reduced endogenous CoQ_10_ level, confirming the negative effect of SARS-CoV-2 on these parameters. Another limitation of the study is that the blood samples from the vaccinated and unvaccinated patient groups were collected at different time points in the COVID-19 pandemic, which could potentially introduce confounding factors to the interpretation of the results. The treatment of the V + PAC-19 group includes information on all drugs used by the patients, which include contraceptives and drugs for the treatment of their chronic diseases. We did not ask the patients from the PAC-19 group for this information. Three patients from V + PAC-19 group and one patient from the PAC-19 group were treated with Isoprinosine, which was considered effective in the treatment of COVID-19. High doses of vitamins C and D, and Zn represented the basis of the treatment for COVID-19 in both groups. Another limitation of the study is the higher average age of the group of unvaccinated patients (PAC19, 59.1 years) in comparison with vaccinated patients (V + PAC19, 43.0 years) and the control group of healthy volunteers (41.3 years). In our previous study, we found slightly reduced CI-linked mitochondrial function and slightly increased CII-linked mitochondrial function in platelets of healthy aged subjects (68.4 years) compared to healthy young subjects (22.6 years). The mean age difference between these groups was 45.8 years, and the differences in platelet mitochondrial respiration were statistically insignificant [74]. The Stronger side of our pilot results is the contribution to elucidating the mechanism of vaccination effect in patients with post-acute COVID-19, which prevents damage to the platelet mitochondrial bioenergetics without the protection of endogenous coenzyme Q_10_ biosynthesis.

## Figures and Tables

**Table 1:** Platelet mitochondrial respiration in vaccinated and unvaccinated patients with post-acute COVID-19 (V + PAC19, PAC19).

O_2_ Flow [pmol/s/10^6^ Cells]	Control n = 16	V + PAC19 n = 10	*p* vs. Control	PAC19 n = 10	*p* vs. Control
ce	0.099 ± 0.008	0.092 ± 0.011	0.6	0.080 ± 0.007	0.13
Dig	0.002 ± 0.001	0.000 ± 0.000	0.13	0.000 ± 0.000	0.13
1PM	0.021 ± 0.003	0.026 ± 0.002	0.18	0.013 ± 0.003	0.073
2D	0.118 ± 0.015	0.108 ± 0.011	0.6	0.075 ± 0.008	0.034
2D;c	0.129 ± 0.016	0.115 ± 0.012	0.5	0.089 ± 0.009	0.026
3U	0.140 ± 0.017	0.119 ± 0.011	0.3	0.092 ± 0.010	0.039
4G	0.161 ± 0.018	0.143 ± 0.013	0.5	0.095 ± 0.011	0.020
5S	0.350 ± 0.027	0.327 ± 0.025	0.6	0.276 ± 0.012	0.071

Legends: see Figure 1; n—number of subjects; Results are expressed as mean ± standard error of mean (sem); *p*—statistical significance vs. control group.

**Table 2:** The concentration of antioxidants in platelets, blood, plasma and concentration of TBARS in vaccinated and unvaccinated patients with post-acute COVID-19 (V + PAC19, PAC19).

	Control n = 16	V + PAC19 n = 10	*p* vs. Control	PAC19 n = 10	*p* vs. Control
Platelets (pmol/10^9^ PLT)					
CoQ_10_	82.4 ± 5.4	65.6 ± 5.3	0.046	58.9 ± 3.6	0.004
α-tocopherol	3666.6 ± 382.1	4556.6 ± 620.4	0.2	1361.1 ± 161.2	0.0001
γ-tocopherol	173.1 ± 33.2	87.9 ± 17.7	0.051	190.5 ± 31.6	0.9
Blood (µmol/L)					
CoQ_10_	0.306 ± 0.023	0.238 ± 0.031	0.085	0.217 ± 0.030	0.031
α-tocopherol	23.3 ± 1.55	21.0 ± 2.08	0.4	18.6 ± 1.99	0.085
γ-tocopherol	1.34 ± 0.24	1.07 ± 0.24	0.4	0.91 ± 0.13	0.2
β-carotene	0.273 ± 0.047	0.330 ± 0.053	0.4	0.302 ± 0.068	0.7
Plasma (µmol/L)					
CoQ_10_	0.475 ± 0.038	0.318 ± 0.038	0.010	0.394 ± 0.043	0.2
α-tocopherol	34.9 ± 2.84	25.3 ± 1.79	0.021	29.4 ± 1.40	0.2
γ-tocopherol	2.17 ± 0.37	1.21 ± 0.19	0.067	1.50 ± 0.17	0.2
β-carotene	0.327 ± 0.054	0.364 ± 0.055	0.7	0.410 ± 0.077	0.4
TBARS (µmol/L)	5.04 ± 0.17	5.42 ± 0.24	0.2	4.62 ± 0.22	0.15

Legends: The concentrations of antioxidants in platelets are in pmol/10^9^ PLT, the concentrations of antioxidants in blood and plasma, and the concentrations of TBARS in plasma are in µmol/L. CoQ_10-TOTAL_ (ubiquinol + ubiquinone); TBARS—thiobarbituric acid reactive substances; Control—control group; V + PAC19—vaccinated patients 2 weeks with post-acute COVID-19; PAC19—unvaccinated patients 4–7 weeks with post-acute COVID-19; n—number of subjects; Results are expressed as mean ± standard error of the mean (sem); *p*—statistical significance vs. control group.

## Data Availability

The supporting data are available from authors upon request.
